# *De novo* whole genome sequencing data of two mangrove-isolated microalgae from Terengganu coastal waters

**DOI:** 10.1016/j.dib.2019.104680

**Published:** 2019-10-18

**Authors:** Kit Yinn Teh, C.L.Wan Afifudeen, Ahmad Aziz, Li Lian Wong, Saw Hong Loh, Thye San Cha

**Affiliations:** a Faculty of Science and Marine Environment, Universiti Malaysia Terengganu, 21030 Terengganu, Malaysia; b Satreps-Cosmos Laboratory, Central Laboratory Complex, Universiti Malaysia Terengganu, 21030 Terengganu, Malaysia; c Institute of Marine Biotechnology, Universiti Malaysia Terengganu, 21030 Terengganu, Malaysia; d Institute of Tropical Aquaculture, Universiti Malaysia Terengganu, 21030 Terengganu, Malaysia

**Keywords:** Next generation sequencing, Oleaginous microalgae, Salinity, IDBA-UD, Chlorophyta

## Abstract

Interest in harvesting potential benefits from microalgae renders it necessary to have the many ecological niches of a single species to be investigated. This dataset comprises *de novo* whole genome assembly of two mangrove-isolated microalgae (from division Chlorophyta); *Chlorella vulgaris* UMT-M1 and *Messastrum gracile* SE-MC4 from Universiti Malaysia Terengganu, Malaysia. Library runs were carried out with 2 × 150 base paired-ends reads, whereas sequencing was conducted using Illumina Novaseq 2500 platform. Sequencing yielded raw reads amounting to ∼11 Gb in total bases for both species and was further assembled *de novo*. Genome assembly resulted in a 50.15 Mbp and 60.83 Mbp genome size for UMT-M1 and SE-MC4, respectively. All filtered and assembled genomic data sequences have been submitted to National Centre for Biotechnology Information (NCBI) and can be located at DDBJ/ENA/GenBank under the accession of VJNP00000000 (UMT-M1) and VIYE00000000 (SE-MC4).

**Table:** Specifications Table

Subject	Molecular Biology
Specific subject area	Whole genome sequencing (WGS)
Type of data	WGS data of: i) *C. vulgaris* UMT-M1 ii) *M. gracile* SE-MC4
How data were acquired	Paired-end sequencing on Illumina Novaseq 2500 platform followed by *de novo* assembly using IUBD-DA
Data format	Raw and filtered *de novo* genome sequences: FASTQ
Parameters for data collection	DNA extracted from axenic cultures
Description of data collection	DNA from fresh microalgae cells was extracted. DNA purity and concentration were measured before sequencing. Data were assembled *de novo* using IDBA-UD assembler.
Data source location	Institution: Institute of Marine Biotechnology, Universiti Malaysia Terengganu City/Town/Region: Kuala Terengganu, Terengganu Country: Malaysia Latitude and longitude (and GPS coordinates) for collected samples/data: i) UMT-M1: 5° 24′ 11.39″ N, 103° 05′ 9.60″ E (Mengabang Telipot, Universiti Malaysia Terengganu) ii) SE-MC4: 5° 31′ 59.2″ N 102° 56′ 52.2″ E (Setiu Wetland, Terengganu)
Data accessibility	Genomes of both species can be found at DDBJ/ENA/GenBank under the accession numbers: i) *C. vulgaris* UMT-M1: VJNP00000000 ii) *M. gracile* SE-MC4: VIYE00000000

**Table:** 

**Value of the Data** • First complete chromosomal genome sequencing of two native microalgae isolated from mangrove area in tropical region. • Further enrich the currently limited WGS data collections of important microalgae species, aid in strain improvement and support interests of various biotechnology industries. • Benefit future works on comparative genome analysis and microalgae adaptation responses.

## 1 Data

Response of microalgae to environmental stimuli is species-specific and may even vary from strain to strain [1,2]. Moreover, mangrove dwelling microalgae are often exposed to impending high and low tides making them unique assemblages in a marginal ecosystem niche with possibly unique responses. Being able to regulate and exert control over the outcome of those responses remain as the most difficult conundrums in phycology research. Both UMT-M1 and SE-MC4 used in this research are oleaginous native species isolated from the mangrove areas in Terengganu, Malaysia. UMT-M1 has been intensively studied in our previous research for oil and fatty acid productions under various culture conditions, such as nitrogen starvation [3], phytohormones treatments [[4], [5], [6]], as well as strain improvement through genetic modifications [7,8]. On the other hand, SE-MC4 is a non-model species which has been observed to produce more than 50% (of dry weight) of total oil content in our laboratory. The exploration on novel genome in a non-model microalga is imperative in order to enrich the available genome data for further biodiesel development applications.

Efforts to improve microalgae feedstock from a molecular aspect is often curtailed by the limited number of available microalgae genomes [9]. Moreover, available *C. vulgaris* genome only constitutes a freshwater species [10]. Following in that prospect, the *de novo* WGS of *C. vulgaris* UMT-M1 featured in this report represents a mangrove dwelling microalga that is able to adapt and survive in a wide range of salinity. Besides that, exploration of potentially high-oil producing non-model species such as *M. gracile* SE-MC4 is pertinent for adding genetic variety to the presently available genetic databank [11].

In UMT-M1, subsequent sequencing generated 73, 495,318 raw reads, amounting to 11,097,793,018 (11.09 Gb) in total bases (Table 1). Overall, 89.58% of total bases achieved a Phred score of Q30 with GC content of 62.29%. High quality raw reads from Table 1 were then filtered, normalized and assembled *de novo* using IDBA-UD assembler [12]. The IDBA-UD assembler internally pipes contigs into scaffolds to form assembled scaffolds. Scaffolds with less than 200 bases were removed. Assembly produced 2547 scaffolds amounting to a total of 50,153,796 bases (50 Mbp). The scaffold positioned at the N50 and N90 were 56,390 and 14,886 bases, respectively (Table 2).

**Table 1:** Statistics of paired-end sequence library for *C. vulgaris* UMT-M1 and *M. gracile* SE-MC4.

Species	Total reads	Total bases	GC Content (%)	Nt* > Q30% (%)
*C. vulgaris* UMT-M1	73,495,318	11,097,793,018 (11.09 Gb)	62.29	89.58
*M. gracile* SE-MC4	72,742,158	10,984,065,858 (10.98 Gb)	68.27	90.52

*Nt = nucleotides.

**Table 2:** D*e novo* sequence statistics for *C. vulgaris* UMT-M1 and *M. gracile* SE-MC4.

Species	Number of scaffolds	Total length (base)	Max length (base)	Min length (base)	N50	N90
*C. vulgaris* UMT-M1	2547	50,153,796	386,660	201	56,390	14,886
*M. gracile* SE-MC4	32,473	60,830,643	52,109	201	2915	802

In SE-MC4, total bases generated from sequencing amounted to 10,984,065,858 bp (10.98 Gb) with 68.27% GC content and a Phred score of 90.52%. Sequencing data statistics are summarised in Table 1. *De novo* assembly in SE-MC4 obtained 32,473 scaffolds and a total length of 60,830,643 bp (60.83 Mb) with maximum length of 52,109 bp and minimum length of 201 bp. Mean length (N50) of scaffolds is 2915 bp, while N90 is 802 bp. Statistics of the genome assembly are as shown in Table 2.

## 2 Experimental design, materials, and methods

### 2.1 Sample preparation

Inoculum stock was obtained from microalgae culture collection at the Universiti Malaysia Terengganu. Stock cultures were maintained under axenic and sterile culture conditions in modified Guillard's F2 medium [3] prepared with artificial seawater (30 ppt). Microalgae cells were harvested at mid-stationary phase. Microalgal cells were harvested from 50 mL of culture by centrifugation at 7000 rpm for 5 min. DNA was extracted from fresh pellet using Wizard® Genomic DNA Purification Kit (Promega, USA). All extraction steps were carried out as per manufacturer's protocol. Prior to sequencing, DNA purity was evaluated via absorbance values of (260/280, 260/230) ratio, gel electrophoresis pattern and double-strand DNA concentration measurements.

### 2.2 De novo WGS sequencing

Library preparation and sequencing were conducted by Theragen Bio Itex, South Korea. Library preparation was carried out using TruSeq Nano DNA Library Prep Kit (Illumina, USA). Library construction was made by DNA size selection attached with adaptors to produce an insert size of 350 bp [13]. Runs were conducted with 2 × 150 base paired-end reads. Sequencing was then performed on Illumina Novaseq 2500 platform. Cluster generation on flow cells was performed by using constructed libraries on cBot equipment (Illumina, USA). Following sequencing of raw reads, adapter sequences were trimmed via cutadapt v1.10 [14] and quality filtering was performed to remove contaminants. Reads that scored above Q30 were selected for assembly. *De novo* assembly of high quality reads was then carried out using IDBA-UD assembler to form scaffolds [12]. Scaffolds that were <200 bp in length were removed manually.

### 2.3 Deposition of genome data

Raw data sequence and assembled genome were deposited in NCBI depository portal. Steps by steps guidelines on submission was followed as in NCBI author guide via https://www.ncbi.nlm.nih.gov/genbank/genomesubmit/. Breakdown of the project accession is shown in Table 3.

**Table 3:** Sequence accession numbers and directory links.

Species	Directory/Data	Accession number	Links
*C. vulgaris* UMT-M1	BioProject	PRJNA550188	https://www.ncbi.nlm.nih.gov/bioproject/PRJNA550188
	BioSample	SAMN12111214	https://www.ncbi.nlm.nih.gov/biosample/SAMN12111214
	Raw sequence (SRA)	SRR9478717	https://www.ncbi.nlm.nih.gov/sra/SRX6245806/
	Assembled genome	VJNP00000000	https://www.ncbi.nlm.nih.gov/nuccore/VJNP00000000
*M. gracile* SE-MC4	BioProject	PRJNA550185	https://www.ncbi.nlm.nih.gov/bioproject/PRJNA550185
	BioSample	SAMN12111213	https://www.ncbi.nlm.nih.gov/biosample/SAMN12111213
	Raw sequence (SRA)	SRR9587833	https://www.ncbi.nlm.nih.gov/sra/SRX6353668
	Assembled genome	VIYE00000000	https://www.ncbi.nlm.nih.gov/nuccore/VIYE00000000
